# A 47-year-old woman with nuclear protein in testis midline carcinoma masquerading as a sinus infection: a case report and review of the literature

**DOI:** 10.1186/s13256-019-2015-x

**Published:** 2019-03-11

**Authors:** Safwan K. Elkhatib, Beth K. Neilsen, Richard L. Sleightholm, Michael J. Baine, Weining Zhen

**Affiliations:** Department of Radiation Oncology, 986861 Nebraska Medical Center, University of Nebraska Medical Center, Omaha, NE 68198-686 USA

**Keywords:** Nuclear protein in testis, *NUT* midline carcinoma, *NUT*–BRD4 fusion, Aggressive tumor, Case report

## Abstract

**Background:**

Nuclear protein in testis midline carcinoma is a rare, highly metastatic undifferentiated carcinoma that typically arises in midline structures and is characterized by having a fusion involving the nuclear protein in testis, *NUT*, gene. Nuclear protein in testis midline carcinoma has been identified in patients of all ages and is often initially misdiagnosed due to the rapid timeline of symptom onset.

**Case presentation:**

Here we report the case of a 47-year-old Caucasian woman with a nuclear protein in testis midline carcinoma that was initially mistaken for a sinus infection. After symptom progression while on an aggressive antibiotic regimen, the source of her symptoms was correctly identified as a sella mass. Comprehensive analysis of the tumor was performed, and standard cytogenetic analysis identified a translocation of 15q and 19p. Further testing identified a *NUT*–*BRD4* fusion and confirmed the diagnosis of nuclear protein in testis midline carcinoma. Despite definitive diagnosis and surgical, radiation, and, ultimately, systemic therapy, she progressed rapidly, developing widespread metastases, and ultimately died from the disease 5 months after diagnosis.

**Conclusions:**

Based on this and other previous reports, aggressive therapy should be initiated once nuclear protein in testis midline carcinoma is diagnosed and close surveillance employed in an attempt to prevent and/or recognize metastases as early as possible. Aggressive therapy has shown little efficacy such that the average overall survival for patients with nuclear protein in testis midline carcinoma is very short, often less than 6 months. Thus, early enrollment into clinical trials testing novel therapies for the treatment of nuclear protein in testis midline carcinoma should be considered. Finally, additional reports of nuclear protein in testis midline carcinoma are needed to fully characterize this rare and highly aggressive cancer.

## Introduction

Nuclear protein in testis midline carcinomas (NMCs) are rare, highly aggressive malignancies that are defined by having genetic rearrangements in the nuclear protein in testis (*NUT*) gene. In a large majority of cases, the *NUT* gene is translocated to form a fusion protein with *BRD4* or *BRD3*. In the remaining cases, known as *NUT*-variant, the *NUT* genes fuse with another binding partner [1]. These tumors typically arise in midline structures and are more commonly found in the head and neck or thorax, but can arise in the abdomen and have even been reported in the medial thigh [2]. There have been several reports of NMC arising in glands within the head and neck including the submandibular gland [3], parotid gland [4], and sublingual gland [5].

NMC often arises in younger patients but has been reported in a wide range of ages from newborns to the elderly (0–78 years) [6, 7]. Characteristically, these tumors exhibit features of poorly or undifferentiated carcinomas [8] and have small round cells on cytopathology [9]. NMC often presents as multifocal disease and is commonly misdiagnosed. In fact, one retrospective study that reviewed the presentation of 12 patients reported that half were initially misdiagnosed [10]. In individual case reports, NMC has been reported to have been initially misdiagnosed as other tumor types including lymphomas [11], adamantinoma [12], primary lung tumors [13], and germ cell tumors [14], which is not surprising as there have been multiple cases reporting positive alpha-fetoprotein (AFP) expression in these tumors [15, 16].

These misdiagnoses are likely, at least in part, to be caused by the unusual patient presentations and rapid onset of symptoms with NMC. In one case report, the patient presented with superior vena cava syndrome [17]. In another case report, a patient presented with a left-sided nasal cavity mass and diplopia [18], and in another case report there were pleural effusions and subsequently identified bilateral ovarian masses [19]. These tumors are also commonly confused with infectious processes with reports describing misdiagnoses of tonsillitis in an 8-year-old boy [20], Epstein–Barr virus (EBV) infection with concomitant *Streptococcus* superinfection in a 23-year-old man [21], and herpes zoster (a cutaneous metastasis of NMC) in a 48-year-old man [22].

NMC is typically a very aggressive cancer with a very short overall survival (average less than 6 months). Patients with *NUT*-variant translocations fare slightly better than those with *BRD4*–*NUT* carcinomas with average overall survival being 96 weeks (*N* = 3) versus 28 weeks (*N* = 8), respectively [1]. However, due to the low incidence of NMC, survival statistics for this disease are generally based on very limited cohorts of patients, and additional case reports are needed to fully characterize NMC and report on the efficacy of different therapeutic regimens.

Here we present the case of a 47-year-old woman who was initially misdiagnosed as having sinusitis. After her symptoms progressed to include nasal burning, severe headache on bending forward, and cranial nerve (CN) VI palsy causing blurred vision 2 weeks later, an undifferentiated mass was identified within her sella turcica that was quickly identified as being NMC. Despite aggressive therapy that demonstrated initial efficacy against the primary tumor, she quickly progressed and died from the disease less than 5 months after diagnosis.

## Case presentation

A 47-year-old previously healthy Caucasian woman was seen at an urgent care clinic for severe headaches and frontal sinus pressure in August. She was prescribed a standard course of amoxicillin-clavulanic acid (Augmentin) and prednisone for a presumed sinus infection. After a week of unimproved symptoms, she was seen by an ear, nose, and throat (ENT) specialist and underwent a rhinoscopy (nasal endoscopy) that revealed edematous nasal passages void of pus. At this time, still believing her symptoms were the result of a sinus infection, her antibiotic was switched to 300 mg clindamycin given three times daily and she was tapered off her steroids (8 mg for 3 days, 6 mg for 2 days, then 4 mg for 2 days, and 2 mg for 2 days). Unfortunately, her symptoms continued to worsen. One week following her endoscopy, she was admitted to the emergency department (ED) for severe headache particularly when bending forward, intermittent left-sided blurred vision, diplopia, and paresthesia (nasal burning). She was started on 750 mg intravenously administered Levaquin (levofloxacin) daily and 80 mg Solu-MEDROL (methylprednisolone) every 8 hours, but the severity of her symptoms and lack of improvement on antibiotics prompted a computed tomography (CT) scan. The CT scan found near-total opacification of the sphenoid sinuses bilaterally in addition to the right maxillary sinus with air-fluid levels with minimal opacification observed in the left posterior anterior ethmoid sinus. While admitted, she developed left-sided CN VI (abducens) palsy and worsening blurry vision that prompted the transfer to our care facility.

Upon arrival to our institution, she was afebrile with stable vital signs and she was ill-appearing, but in no acute distress: temperature (T) 36.7 °C, heart rate (HR) 49 beats per minute, blood pressure (BP) 132/68 mmHg, and respiratory rate (RR) 16 breaths per minute. A physical examination revealed a normal physical examination. A neurological examination and portable slit lamp examination were also within normal limits with the exception of symptoms from the left CN VI palsy. Thorough laboratory studies were completed upon admission including metabolic panels, complete blood counts, urine analysis (UA), microbiology, and serology. Her white blood cell count was elevated upon presentation (21.9 × 10^9^ cells/L), trended downwards to 13.9 × 10^9^ cells/L within 5 days, and remained at a slightly elevated level for the next several months. Inflammatory markers C-reactive protein and lactate dehydrogenase were elevated upon presentation, but erythrocyte sedimentation rate and procalcitonin were within normal limits. Her remaining laboratory results were within normal limits or negative: comprehensive metabolic panel (CMP), Mg, phosphorus (Phos), UA, antinuclear antibodies (ANA) panel, antineutrophil cytoplasmic antibody (ANCA), prolactin, thyroid-stimulating hormone (TSH), free thyroxine (T4), cortisol, growth hormone (GH), follicle-stimulating hormone (FSH), luteinizing hormone (LH), insulin-like growth factor 1(IGF-1), arterial blood gas (ABG), pregnancy, and HIV. In regards to microbiology, a Gram stain from nasal and maxillary sinus demonstrated white blood cells, but no organisms were present except for rare normal respiratory tract flora. Aerobic, anaerobic, and fungal cultures from these specimens demonstrated no growth. Fungitell®, *Aspergillus* galactomannan antigen, and *Toxoplasma gondii* antibody tests were all negative.

**Fig. 1:**
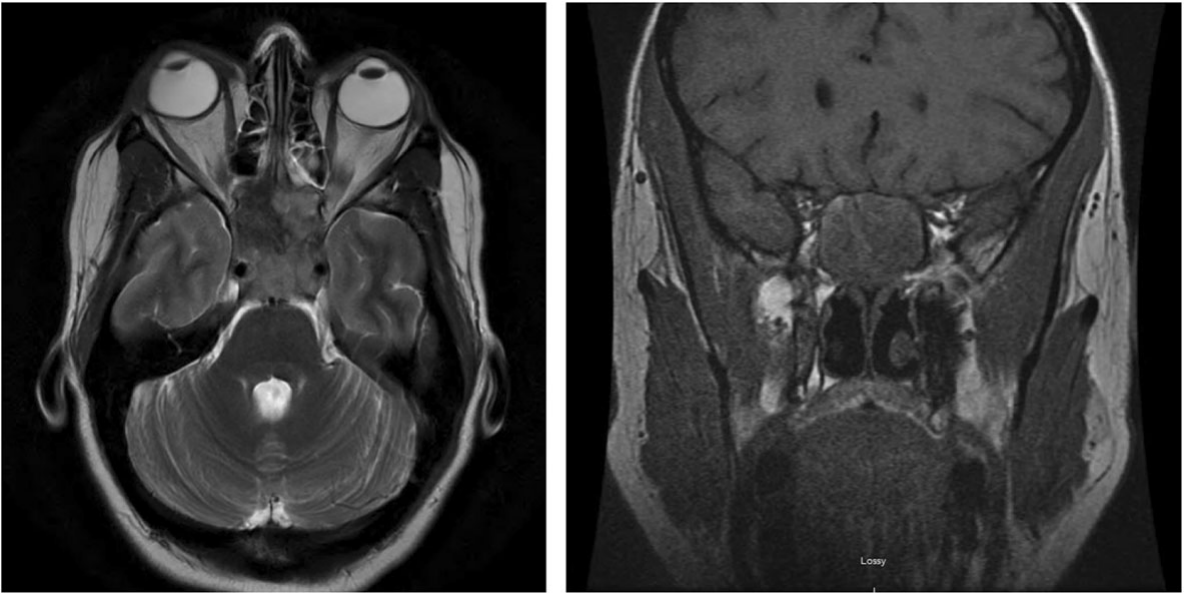
Magnetic resonance imaging demonstrating the identified 2.6 by 1.7 cm mass centered in the sella extending superiorly along the pituitary infundibulum, laterally into the cavernous sinuses, bilaterally to abut the carotid arteries, and anteriorly into the bilateral sphenoid sinuses with transverse (*left*) and coronal (*right*) planes shown

Upon transfer to our institution, the previous CT scan findings prompted an immediate follow-up magnetic resonance imaging (MRI) of her head with and without contrast that included orbits; the MRI revealed a 2.6 by 1.7 cm mass centered in the sella extending superiorly along the pituitary infundibulum, laterally into the cavernous sinuses, bilaterally to abut the carotid arteries, and anteriorly into the bilateral sphenoid sinuses (Fig. 1). Transnasal transsphenoidal excision and debulking was performed for what was thought to be an invasive pituitary macroadenoma. Intraoperatively, the mass was noted to be firm, rubbery, ill-defined, infiltrative with protrusion through the sellar face, and eroding through the clivus. Due to the infiltrative nature of the tumor, a complete resection was not possible. Of note, during resection, the mass appeared to be extradural and separate from the pituitary gland. Consistent with this, frozen tissue sections obtained during surgery were concerning for malignancy but did not appear to be a pituitary adenoma.

An initial pathologic inspection was suggestive of melanoma, however, further pathologic analysis over the next few weeks suggested this was not the case. Specimens were diffusely positive for SOX10 and CD99, focal positivity of S100, but failed to stain the following markers: cytokeratin, AE1/AE3, CAM5.2, Melan-A, synaptophysin, CD45, CD20, Pax5, ERF, Pax8, Gata3, HMB45, MiTF, cytokeratin 7, CD56, calretinin, and chromogranin. Simultaneous cytogenetic testing showed a reciprocal translocation involving 15q and 19p, which led to the addition of NMC to the differential [23]. Further fluorescence *in situ* hybridization (FISH) testing confirmed the fusion of *NUTM1* (15q14) and *BRD4* (19p13.12) loci confirming the diagnosis [23].

In attempt to determine if our patient had any notable exposures that could have contributed to the development of this rare disease, a full social and environmental history was solicited including evaluation of relevant exposures. She lived with her spouse, two dogs, and a cat in a suburban, recently built home. She worked for the suburban public-school system largely ruling out work or home exposures. She also denied any use of tobacco, alcohol, or illicit drugs. Our patient and her family denied any unusual chemical or toxic exposures. Our patient’s family history is positive for hyperlipidemia, stroke, and lung cancer in our patient’s mother (died at age 69) and hyperlipidemia, hypertension, and testicular cancer in our patient’s father. Our patient’s brother and her three children (one daughter, two sons) are all alive and without any known medical issues.

**Fig. 2:**
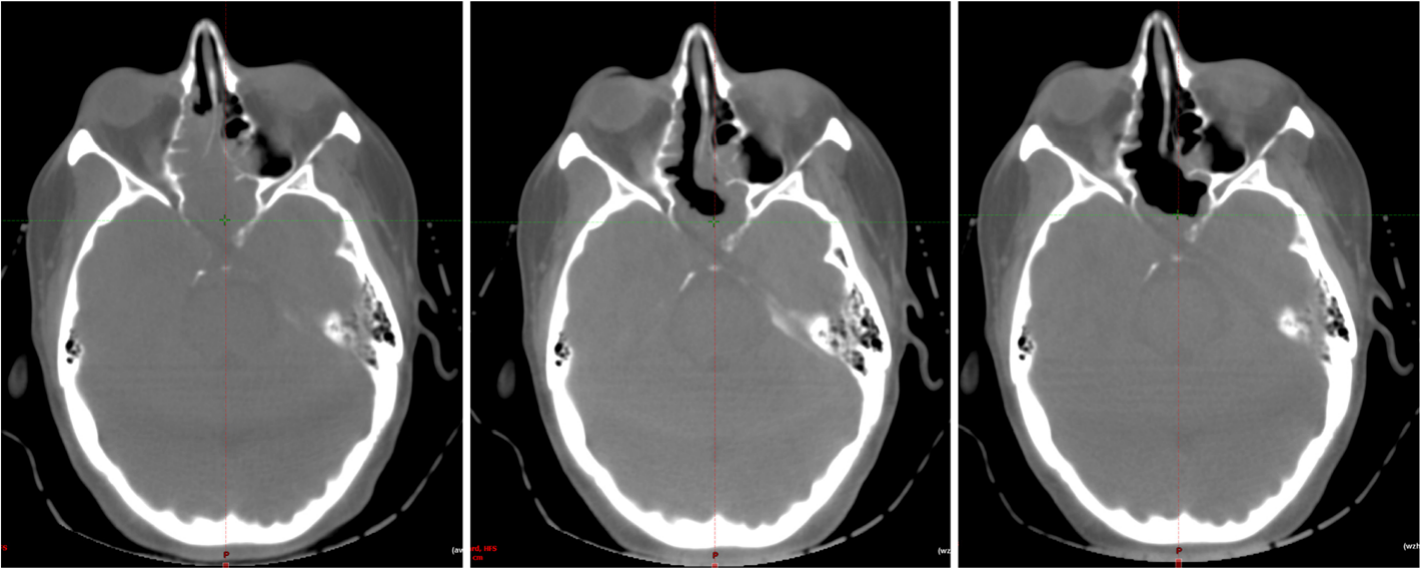
Primary tumor response during radiation therapy as demonstrated by cone-beam computed tomography from first (*left*), tenth (*middle*), and final (*right*) fractions

Due to the initial consideration that our patient’s tumor may represent melanoma, she underwent a whole-body positron emission tomography (PET)-CT scan to search for additional sites of tumor involvement. The sphenoid region displayed a significant uptake in addition to two left-sided, level-2 lymph nodes. Ultrasound of her neck found a single slightly enlarged lymph node measuring 1.1 cm with fatty hilum that was thought to be the source of increased glucose uptake. At this time, this was not thought to represent malignant involvement. The following week, our patient initiated radiation (50 Gy in 20 fractions) to her sella, cavernous sinuses bilaterally, and skull base. Over the course of radiation therapy, the primary tumor dramatically decreased in size suggesting it was highly radiosensitive (Fig. 2). Unfortunately, however, at the initial radiation treatment, the neck nodes were found to have substantially increased in size, and neck CT demonstrated rapidly increased bilateral necrotic nodes that were now believed to represent an unusually rapidly progressive neoplastic process. In addition, a cluster of pulmonary opacities was visualized that were suspicious for metastatic disease. She was then started on combined docetaxel (160 mg, 75 mg/m^2^) and cisplatin (161 mg, 75 mg/m^2^), four total cycles, with concurrent bilateral neck radiation treatment of 50 Gy in 20 fractions.

**Fig. 3:**
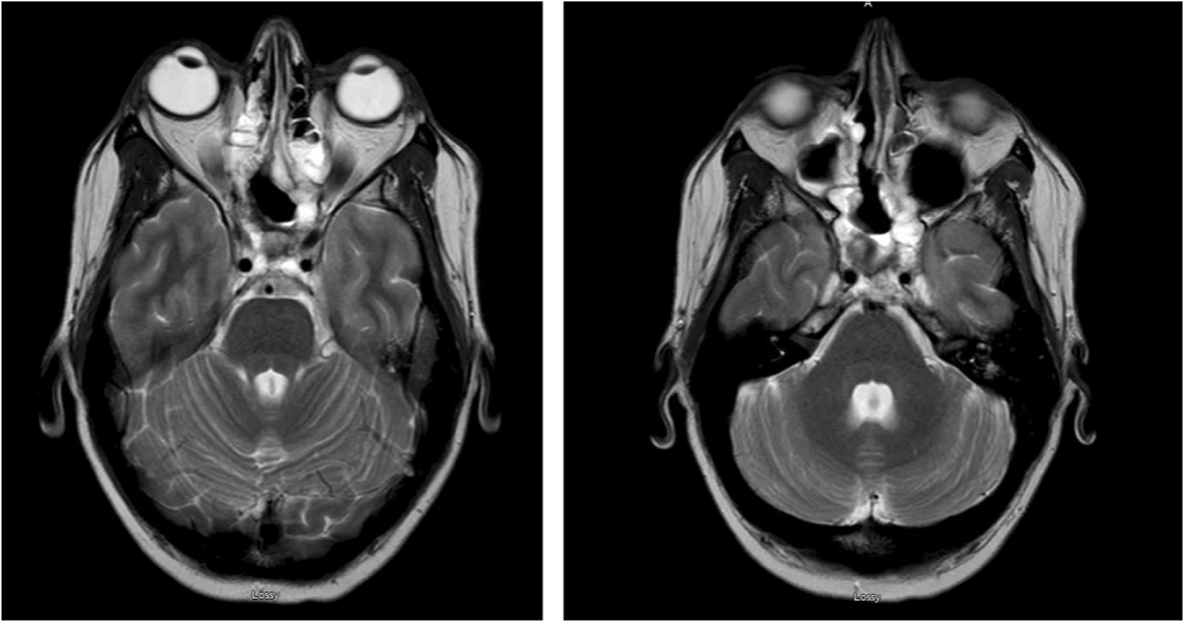
Magnetic resonance imaging demonstrating significant primary tumor response 2 months after resection and status post-initial chemoradiation therapy (+ 69 days from Fig. 1)

Two months after resection and after the completion of concurrent chemoradiation therapy (69 days from MRI shown in Fig. 1), an MRI showed a significant decrease in skull base/sinus lesion (Fig. 3). However, she presented once again to the ED approximately 1 month after cessation of therapy with complaints of pleuritic chest and mid-back pain. A chest CT scan identified a distinct 4 mm pulmonary nodule in the upper lobe of her right lung that was not visualized 3 months prior. An abdominal-pelvic CT scan found bulky retroperitoneal soft tissue masses with severe, proximal inflammatory stranding, suggestive of diffusely metastatic disease. A pain plan was put in place and out-patient endoscopic ultrasound (EUS)/endoscopic retrograde cholangiopancreatography (ERCP) was scheduled for further disease assessment, but increased mid-back pain and refractory constipation prompted a return to the emergency room (ER) before these studies could be completed. At that time another abdominal-pelvic CT scan revealed progression after only 1 week and now demonstrated progressive intra-abdominal lymphadenopathy (9 cm greatest involvement) with encasement of the celiac axis, hepatic and splenic arteries, superior mesenteric vein (SMV), portal vein, gastroduodenal artery with extensions into the pancreatic tail, and compression of the second and third portions of the duodenum. Fine needle aspirate of the pancreas confirmed the presence of metastatic disease, demonstrating malignant cells that were cytomorphologically similar to the original tumor samples. She was admitted for pain control and underwent the EUS/ERCP to further evaluate her metastatic disease. After developing an improved pain control plan, she was discharged.

Unfortunately, after she was released from our hospital, she required readmission less than a week later for intractable nausea and vomiting. Repeat imaging (abdominal-pelvic CT) demonstrated the numerous abdominal masses had increased in size with near-complete occlusion of the SMV and portal vein, increased encasement and narrowing of the celiac artery and superior mesenteric artery (SMA), and significant extrinsic compression of the second and third portions of the duodenum with probable tumor invasion/obstruction. After management of her symptoms, she was once again released from our hospital. She went on to receive palliative radiation to her abdomen (37.5 Gy planned in 15 fractions but she only received 22.5 in 9 fractions). She was readmitted to our hospital following a visit to our ER for recurrent intractable nausea/vomiting, shortness of breath, and general decline. She was transferred to our intensive care unit (ICU) following a hypoxic event and cardiac arrest that required intubation and resuscitation. This was preceded by vomiting and likely aspiration including oral secretions. At this time, a chest X-ray demonstrated persistent low lung volumes and increasing bilateral patchy pulmonary opacities concerning for worsening pulmonary edema in addition to new bilateral pleural effusions and significant ascites. In addition, she demonstrated signs of being critically ill with multiorgan failure. She continued to receive supportive and palliative care until she died from the disease almost 5 months after diagnosis. Since a definitive diagnosis had been established for our patient and in accordance with her family’s wishes, no autopsy was performed.

## Discussion

### Initial patient presentation

Unfortunately, our patient was initially misdiagnosed as having sinusitis, given antibiotics, and sent home. Her symptoms soon progressed and, ultimately, she ended up in our ER and was admitted. In addition, even after her tumor was recognized, it was mistaken for melanoma, and it took nearly 3 weeks for it to be identified as a NMC. This case reinforces the point that in young patients with undifferentiated or poorly differentiated tumors involving midline structures, the clinical suspicion for NMC should be high and testing for *NUT* translocations and/or *NUT* expression should be performed. While the prevalence of this tumor is unknown due to its rarity and relatively recent discovery, between 2 and 18% of tumors that were specifically tested were identified as being NMC in retrospective studies [7, 24–29]. These percentages vary significantly due to relatively small cohort sizes and differences in initial selection criteria, but all included poorly or undifferentiated carcinomas in midline structures. It is important to identify patients with NMC because recent case reports have shown some efficacy with targeted therapies in NMC.

### Therapy

Due to the small number of patients with NMC, no standard therapy regimen has been established. In addition, these patients are often not identified as having NMC until after therapy has been initiated. Therefore, patients often undergo highly individualized therapeutic regimens. Overall, patients tend to undergo surgical removal if they have a single localized mass, while patients with multifocal disease immediately start chemotherapy. Several groups have previously reported treating patients with NMC with intensive chemotherapeutic regimens typically in conjunction with radiation [30–34].

Our patient underwent incomplete surgical resection of her initial sellar mass followed by radiation to the primary site, and subsequently developed neck masses, in conjunction with four cycles of docetaxel and cisplatin. In our patient, radiation therapy appeared to be efficacious against the primary tumor and initial neck metastases; however, she rapidly progressed and developed widespread metastatic disease such that it is difficult to truly assess the effectiveness of the radiation therapy. However, our experience suggests radiation may be efficacious in local control, but due to the aggressive nature of NMC and rapid development of metastatic disease, systemic therapies are needed at the onset of treatment.

Further, the addition of chemotherapy when our patient first demonstrated metastatic disease was unable to prevent the further development of metastases. This is consistent with other reports of NMC that depict the natural history of this disease as having a good response to therapy in the primary tumor followed by a short (1–4 months) period of remission. This is followed by the development of aggressive, widespread metastatic disease that fails to respond to therapy resulting in the rapid decline of the patient. Our patient was offered participation in a phase 1 trial after failing traditional therapies; however, she declined due to her substantially advanced disease at that point. Overall, the hastened timeline of this disease suggests that early aggressive therapies are needed. Therefore, enrollment in clinical trials at the time of diagnosis instead of waiting for metastases may be warranted.

Based on research demonstrating that mechanistically *NUT*–*BRD4* fusion proteins alter chromatin modifications, both bromodomain and extra-terminal motif (BET) [35] and histone deacetylase (HDAC) [36] inhibitors have been tested and shown some efficacy against NMC [37].

In one patient that initially responded to combination chemotherapy, yet underwent rapid disease progression and the development of metastasis, treatment with vorinostat (HDAC inhibitor) provided an objective response. Unfortunately, the patient still died less than a year after her initial diagnosis [38]. In another patient, after no signs of improvement from two cycles of cisplatin, ifosfamide, and etoposide, the patient was enrolled in a BET inhibitor trial (GSK525762, NCT01587703); however, the patient progressed too quickly to see any benefit of the targeted therapy [39]. These cases highlight the potential to treat NMC with targeted therapies, and further reinforce the need to quickly identify NMC and pursue aggressive treatment options early.

Additional basic research had shown that NMC may also be susceptible to CDK9 [40–42] or MYC inhibition [43, 44], but further studies are needed to validate these findings. As we learn more about the mechanism of action behind the *BRD4*–*NUT* fusion protein, additional therapies could be identified and/or developed that may be efficacious in treating this highly aggressive tumor type.

### Follow-up

Consistent with previous reports of NMC, PET-CT scanning revealed increased uptake in the primary tumor as well as in the metastases suggesting this modality could be useful in identifying metastases earlier, which may be critical in selecting therapies and trying to stay ahead of this aggressive tumor. Similarly, follow-up imaging probably should occur soon after completion of therapy (~ 1 month) and at short intervals thereafter due to the aggressive nature of NMC.

### Disease progression/metastasis

The natural history of this disease is that it usually stays relatively localized until the primary tumor is treated, either surgically or with radiation; however, then the patient typically relapses with aggressive metastatic disease. In addition, the primary tumor is often radiosensitive and sometimes chemosensitive, but the metastases are highly resistant. It is difficult to confirm this resistance though as the aggressive nature of the disease often leads to the patient’s death before we can reasonably assess response. However, this perceived differential response to treatment across primary and metastatic disease may be indicative of some type of difference in the biology of the primary tumor versus metastases. After surgical and radiological intervention, our patient quickly developed widely spread metastases. Another possibility is that treatment of the primary tumor inadvertently allows for or promotes seeding of other sites with cancer cells. Alternatively, it is possible that the primary tumor exerts some type of growth control over the metastases and after being removed, this restriction is released. Finally, this observation could simply be characteristic of the natural history of NMC as the average overall survival for patients is very short (6–9 months) with the primary tumor growing rapidly, initially prompting diagnosis and local treatment, but with preceding or simultaneous seeding of metastatic sites occurring such that it is only a matter of time before widespread metastases develop, often even in the presence of systemic chemotherapy.

Finally, the distribution of metastases in our patient was thought-provoking. Her tumor was initially identified in the sella turcica; it metastasized to lymph nodes in her neck but ultimately spread throughout her abdomen. Based on imaging, the abdominal metastases probably started within the celiac/SMA lymph nodes and then spread to seed the entire peritoneal cavity. Interestingly, it appears as though her metastases may also have had a predilection for midline sites. This opens the possibility that the initial tumor identified in our patient within the brain may not have been her “primary” at all, but the first metastasis to develop whereas the actual primary tumor went unnoticed and continued to seed other sites of metastasis.

## Conclusions

NMC is a rare and highly aggressive cancer. In rapidly developed undifferentiated carcinomas, particularly in younger patients, the clinical suspicion for NMC should be high. Once the diagnosis is confirmed, aggressive therapy is needed, and systemic therapy, or even clinical trials, should be considered even in the context of a single primary tumor being evident. Due to the natural history of disease including rapid development of widespread metastatic disease, baseline imaging, close follow-up, and high suspicion for metastatic development should also be applied in these patients.
